# A simple, automated device for the precise addition of liquids

**DOI:** 10.1155/S1463924689000015

**Published:** 1989

**Authors:** Gary W. Kramer, Jose M. Hanquier, A. Roger Frisbee, Philip L. Fuchs

**Affiliations:** Department of Chemistry Purdue University West Lafayette Indiana 47907 USA

## Abstract

Metering liquid reagents into reaction mixtures in a controlled and reproducible manner has often been a problem in synthetic chemistry. Carrying out the real simultaneous addition of two or more liquid reagents (concurrent additions) is even more inconvenient. Difficulties increase when addition volumes become small, when addition times become long, or when the reagents are corrosive or air-sensitive. We have constructed and tested an inexpensive, automated device for the slow, precise delivery of liquid reagents into laboratory-scale reaction mixtures. Controlled by a standard personal computer, this slow adder can accommodate liquid volumes from hundreds of microlitres to litres and addition times from minutes to days. Its glass and Teflon construction makes it useful for nearly all reagents. By using multiple slow adders, true concurrent addition of several liquids can be easily achieved.


Journal of Automatic Chemistry Vol. 11, No. 1 (January–February 1989), p. 1–14

### A simple, automated device for the precise addition of liquids

**Gary W. Kramer†, Jose M. Hanquier, A. Roger Frisbee and Philip L. Fuchs**

*Department of Chemistry, Purdue University, West Lafayette, Indiana 47907, USA*

*Metering liquid reagents into reaction mixtures in a controlled and reproducible manner has often been a problem in synthetic chemistry. Carrying out the real simultaneous addition of two or more liquid reagents (concurrent additions) is even more inconvenient. Difficulties increase when addition volumes become small, when addition times become long, or when the reagents are corrosive or air-sensitive. We have constructed and tested an inexpensive, automated device for the slow, precise delivery of liquid reagents into laboratory-scale reaction mixtures. Controlled by a standard personal computer, this slow adder can accommodate liquid volumes from hundreds of microlitres to litres and addition times from minutes to days. Its glass and Teflon construction makes it useful for nearly all reagents. By using multiple slow adders, true concurrent addition of several liquids can be easily achieved.*

The slow, constant introduction of liquids into reaction mixtures has long been a problem in laboratory-scale organic syntheses. The rate of reagent addition is often a critical variable in determining the outcome of a reaction. However, addition rates are usually specified in nebulous terms such as 'the solution is added dropwise over an hour' or 'the material was added slowly over a period of 5 min'. These statements seem to imply that material was added at a linear rate during the prescribed interval. In actual practice this is rarely the case since precise additions are difficult with the apparatus commonly employed. Failure to account for rates of reagent addition can lead to irreproducibility in experimental results.

Traditional devices such as 'constant addition' funnels, even when fitted with stopcocks containing needle valves, are difficult to adjust to provide constant addition rates. Syringe pumps and dispensers permit easy flow rate control, but suffer from 'freeze up' problems when used with air-sensitive reagents or solutions of solids dissolved in volatile liquids. The volume deliverable from syringe pumps without resetting is limited to 100 ml. Metering pumps, while usable with large volumes, are not suitable for delivery of small amounts of expensive reagents.

Continuing development of an automated synthesis system [1] rekindled the authors' interest in a device for the slow addition of reagents which could be controllable by a general-purpose computer. The current apparatus (figure 1) is an adaptation of a logic-controlled device developed several years ago for bench-scale reagent additions [2]. The equipment consists of a pressurized reagent reservoir, a computer-controlled solenoid valve, and a liquid presence detector. By accurately controlling the time that the valve is open, the rate at which the valve is opened, and the pressure drop across the system, a precise delivery of reagent can be achieved.

† Author to whom correspondence should be addressed.

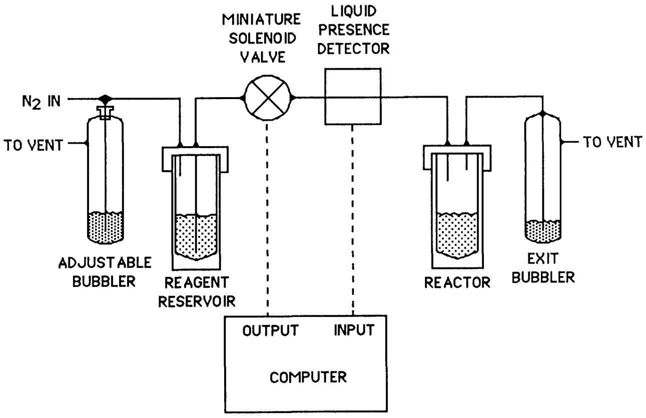

*Figure 1. The slow adder apparatus for liquid additions.*

The heart of the slow addition device (slow adder) is a miniature, Teflon solenoid valve which has a dead volume smaller than 50 μl and an activation time about 8 ms [3]. The valve driver is an electronic device which converts the computer output signal into a precisely controlled, high current drive for the valve. A potentiometer adjustment on the valve driver is used to set the open-time of the valve [4]. The adjustable bubbler [5], used to set the pressure drop across the system, must provide a pressure slightly higher than that of the exit bubbler so that liquid will flow from the reservoir to the reactor when the valve is open. The liquid presence detector [6], used to determine the beginning and end of addition, as well as certain error conditions, should be placed as close as possible to the reactor. The system plumbing is 0·063 in or 0·125 in OD Teflon or Teflon-FEP tubing with ChemInert flare fittings. In the standard system, a reagent comes in contact only with glass and Teflon.

The valve driver and liquid presence detector are designed to work with TTL level signals such as those normally found on a computer's parallel I/O ports. An additional interface must be employed to allow the slow adder to use the RS-232 levels typically found on a computer's serial I/O port [7].

The rate of reagent addition is determined by several variables:

(1) The pressure drop from the reservoir to the reactor.

(2) The length of time that the valve is open.

(3) The rate at which the valve is opened.

0142–0453/89 $3.00 © 1989 Taylor & Francis Ltd.

(4) The length and inside diameter of the delivery tubing.

(5) The viscosity of the liquid being transferred.

The apparatus is assembled as shown in figure 1. The pressure differential between the reservoir and the reactor is adjusted along with the valve open-time to produce the required 'drop size' [8]. A simple computer program pulses the valve open and closed while these manual adjustments are made. For accurate work, the system must be calibrated under conditions as close as possible to those of the actual delivery. A known volume of the reagent is placed into the reservoir, and the valve is pulsed until the liquid presence detector senses the liquid [9]. The computer then counts the number of pulses issued until the detector signals no more liquid. A calibration factor is calculated from the known volume of reagent and the number of pulses required.

The computer program [10] for a controlled addition required the calibration factor, the desired addition time, and the volume of reagent to be added. When these constants are provided, the valve is pulsed rapidly until the detector senses liquid, then the pulse rate is adjusted to that required to give the desired addition time. The program monitors the total addition time and returns an error message if the addition is not complete in 120% of the allocated time. When the reagent is completely added, the true addition time is reported.

The utility of the slow adder can be extended by driving two or more valve drivers from the same computer signal. If each valve subsystem is properly calibrated, a true simultaneous addition of several reagents can be achieved. For best results the reservoirs of each valve system should be pressurized from the same adjustable bubbler. The delivery tubing of each system should be as identical as possible. The reagent concentrations and solution viscosities should be equal. By listening carefully to the clicking of the valves and adjusting the potentiometers until only a single click is heard, the open-time of each valve can be made coincident. The precision of this aural method appears to be equal to that achieved by using an oscilloscope to view the drive signals.

The applicability of the slow adder system is not limited to simple timed reagent additions. By incorporating other computer-readable sensors, such as reaction temperature or pH probes, the computer can be programmed to deliver reagents at rates dependent on other factors. A more sophisticated computer program than the timed addition routine would be required to control such closed loop processes.

Our initial slow adder qualification tests consisted of metering in 1 ml aliquots of liquids (THF, $Et_2O$, $CH_2Cl_2$, hexane, toluene, and water) over intervals ranging from 5 min to 10 h. In addition, we transferred several 100 ml portions of THF over 1 to 3 h. In all cases, the true addition time was within ± 8% of the requested time. We were able to demonstrate the suitability of the slow adder for use with air-sensitive materials by delivering 1 ml aliquots of a solution of 0·10 M MeLi in $Et_2O$ with no change in titre.

To more fully understand the operation of our slow adder, we carried out detailed studies on addition of six liquids ($Et_2O$, hexane, THF, water, EtOH, and a 50:50 by weight mixture of ethylene glycol : water). In each study, the volume delivered was 4·0 ml, the requested delivery time was 30 min, the delivery pressure was 20 mm Hg, and the valve open-time was 100 ms. The calibration factor was obtained from a previous run using a 3 ml volume of the liquid. The apparatus was assembled much as shown in figure 1, except that the reactor and exit bubbler were replaced by a receiving flask placed on a computer-readable balance. The receiving flask was constructed from a 50 ml volumetric flask with its neck replaced by a 4 mm ID tube, 65 mm long, through which a 100 mm 19-gauge syringe needle delivered the liquid without touching the sides. This design was chosen to minimize solvent evaporation in the open flask during the tests. The solvent reservoir was a 5 ml Wheaton vial fitted with a 66 mm 19-gauge syringe needle with a flat-cut tip leading directly to the bottom. The delivery tubing was 1·0 mm ID and was connected to the valve with ChemInert flare fittings and to the syringe needles with Kel-F Luer to Chem-Inert adapters. Two configurations of the slow adder, which differed only in the length of the interconnecting tubing, were examined. The short system incorporated 380 mm of tubing while the long system had 775 mm of tubing.

The results of these studies are shown in table 1. Figure 2 shows the delivery of water in both the long and short system. Delivery of the other liquids produced similar graphs. In each of the curves, certain regions can be identified. Points in the base region labelled 'A' occur while the liquid is filling the delivery tube and has not yet reached the balance. The linear region, 'B', represents the time when both the delivery tube and the reservoir contain liquid. The curved section, 'C', is produced when the reservoir is empty, and the delivery tube is being drained. Finally, the flat region, 'D', shows that the delivery is complete.

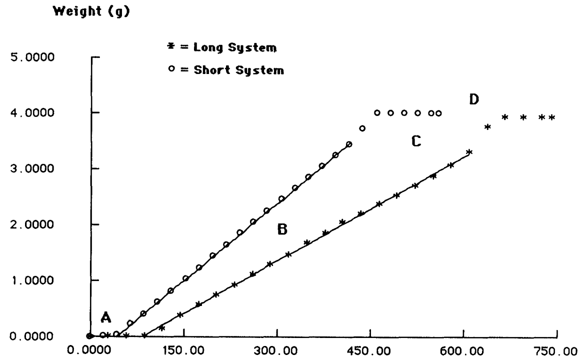

*Figure 2. The addition of water using the slow adder.*

A simple model of the laminar flow of liquid in a horizontal tube shows that the volume flow rate should be directly proportional to the pressure differential across the tube and proportional to the fourth power of the tube's inside diameter, but inversely proportional to the viscosity of the fluid and to the length of the tube (see Appendix 1.D) [11]. The slow adder is a more complicated system than a simple tube, and violates several of the assumptions used to derive the simple flow model.

*Table 1. Slow adder performance.*

| *Liquid* | *Cal. Factor pulses/ml* | *Slope mg/pulse* | *% RSD Slope** | *% Total delivery Time deviation* |
|---|---|---|---|---|
| *Short system* (tubing volume 300 μl, total dead volume 665 μl) | | | | |
| $Et_2O$ | 47 | 12·4 | 0·14 | −4·7 |
| *n*-$C_6$ | 55 | 10·9 | 0·26 | −2·7 |
| THF | 70 | 12·2 | 0·19 | −4·7 |
| $H_2O$ | 112 | 9·2 | 0·30 | −3·3 |
| EtOH | 118 | 6·4 | 0·21 | −0·9 |
| Glycol** | 274 | 4·3 | 0·50 | −8·0 |
| *Long system* (tubing volume 610 μl, total dead volume 975 μl) | | | | |
| $Et_2O$ | 73 | 8·8 | 0·21 | −3·8 |
| *n*-$C_6$ | 73 | 7·8 | 0·22 | −0·2 |
| THF | 125 | 7·3 | 0·19 | −6·7 |
| $H_2O$ | 148 | 6·2 | 0·72 | +5·1 |
| EtOH | 153 | 4·8 | 0·19 | +2·3 |
| Glycol** | 321 | 3·1 | 0·23 | +5·8 |

Conditions: 3 ml calibration volume, 4 ml delivery volume, 30 min requested delivery time, 20 mm Hg delivery pressure, 100 ms valve open-time.

* Linear portion of delivery curve
** Ethylene glycol : water 50*50 by weight.

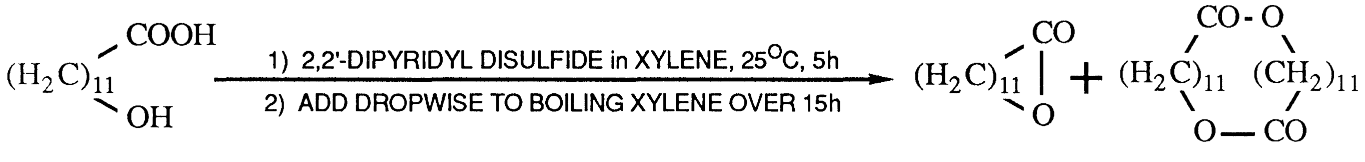

**1** **2** **3**

*Scheme 1. Cyclic lactonization of w-hydroxyalkylcarboxylic acids using the slow adder.*

However, it is reassuring that the slopes of the linear region of the addition curves (which represent flow rates) show inverse, though not completely linear, dependencies on both liquid viscosity and tube length. Also the shape of the flow curves in region C can be derived from the flow model (see Appendix 1.D).

The shape of the delivery curves affect the delivery time accuracy of the slow adder. Calibration is usually done by placing a known volume of the liquid in the reservoir and then counting the number of pulses required to totally dispense it. Starting and stopping the pulse counting is done on signals from the liquid presence detector mounted as close as possible to the reactor. The calibration factor is calculated by dividing the number of pulses required by the volume delivered. The increase in rate that occurs while the delivery tube is drained causes an error in the calibration. This error is exacerbated by longer tube lengths which cause the C region of figure 2 to become larger. Minimizing the lengths of the tubing between the reservoir and the reactor will reduce the error, but since the slow adder is a 'to contain' device, it will always be present. The use of small calibration volumes can also lead to larger errors – the number of pulses in the B region decreases in relation to the number of pulses in the C region.

A better calibration factor can be obtained by either counting the number of pulses required to deliver a certain weight of liquid onto a balance or to deliver a certain volume to a volumetric measure when the slow adder is in the linear region of its delivery curve. However, these methods require either additional automated equipment or human intervention.

To provide a complete application of the slow adder, a reported lactonization procedure [12] was carried out (Scheme 1) – reagent addition had been done in this over 15 h using a mechanical syringe drive. A mixture of 12-hydroxydodecanoic acid 1 (0·108 g, 0·5 mmol), 2,2′ dipyridyl disulfide (0·165 g, 0·75 mmol) and triphenylphosphine (0·197 g, 0·75 mmol) was dissolved in dry, oxygen-free xylene and stirred at 25 °C for 5 h. The slow adder was calibrated by adding 10 ml of xylene from the reservoir to the reactor containing 90 ml of xylene at reflux under nitrogen (calibration factor: 0·008 ml/valve pulse). The solution of hydroxyacid 1 was transferred to the addition reservoir and added under computer control to the boiling xylene (specified addition time = 15·0 h, actual addition time = 16·1 h). The addition reservoir was rinsed with 1 ml xylene, which was flushed into the reactor. After heating at reflux for an additional 10 h, the solvent was removed under reduced pressure and the ether-soluble portion was purified by silica gel chromatography using 2% THF-hexanes to yield 0·075 g (0·379 mmol, 76%) of the desired lactone 2 (reference yield 66% after recrystallization). The product identity was confirmed by spectral comparison to an authentic sample [13].

To demonstrate the utility of the device for true concurrent additions, two valves were driven from one input signal. Two 100 ml reservoirs were pressurized from the same adjustable bubbler (about 15 mm Hg). The valve open-times were set aurally and verified by oscilloscope measurement of the drive signals (time open about 50 ms). The repetition rate was set at one drop every 5 s. Delivery tubes consisted of identical 18-gauge syringe needles with 18 in cannulae leading from the reservoirs to the valves and from the valves to an open 150-ml beaker (reactor). One reservoir was charged with 0·102 M HCl solution and the other was filled with 0·102 M NaOH solution. The concentrations of the acid and base were painstakingly adjusted to be equal. The beaker was fitted with a magnetic stirring bar, about 10 ml of water, and a few drops of methyl purple indicator [14]. Stirring was begun, and the colour of the liquid in the beaker was adjusted by dropwise addition of acid and base to give the neutral grey colour. The simultaneous addition of the acid and base was begun. Green and purple flashes of colour could be seen in the beaker as addition proceeded, but the overall grey liquid colour was sustained until over 25 ml of acid and base had been added. A purple tinge then began to develop, and the addition was terminated. Less than one drop of the acid or base was required to completely change the colour of the liquid in the beaker from grey to violet or green.

Electrical schematics for the valve driver, liquid detector, optional RS-232 interface, mechanical specification for the liquid detector, a derivation of a simple liquid flow model, and a source listing of a sample timed addition program are found in Appendix 1.

### Acknowledgement

This development was supported by a grant from the National Science Foundation (CHE-8406115).

### References and notes

1. For recent developments see: Kramer, G. W. and Fuchs, P. L., In *Advances in Laboratory Automation Robotics 1986*; Hawk, G. L. and Strimaitis, J. R. (Eds) (Hopkinton, MA, 1986), p. 361.
2. Kramer, G. W., unpublished results, 1976.
3. The Series 2 two-way valve is available from General Valve Corporation, 202 Fairfield Road, Fairfield, New Jersey 07006, USA.
4. Valve open-times are adjustable from 16 to 512 ms. Hardware control of the valve open-time, although not essential since the computer can generate accurate time delays, simplified the programming. To eliminate the possibility of the computer trying to pulse the valve driver during the time the valve is already open and thereby losing a pulse, the software sets 1 Hz as the fastest valve pulse rate. If it is known that the valve open-time is near the short end of its range, the software can be modified to allow faster pulsing as long as the inverse of the pulse rate is longer than the valve open time.
5. The adjustable bubbler, P/N 8762-14 is available from Ace Glass Inc., 1430 Northwest Boulevard, Vineland, New Jersey 08360, USA.
6. The liquid sensor has been described in detail elsewhere Kramer, G. W. and Fuchs, P. L. *BYTE*, **11** (1), (1986), 263.
7. The RS-232 control signals DTR and DCD are used to send the valve output pulse and to receive the detector input. If the control signal to the valve driver is derived from a switch or relay contact closure, it must be thoroughly debounced to avoid multiple triggering of the valve driver.
8. When the delivery line between the valve and the reactor is short, the valve closing action ejects the 'drop' into the reactor. Under these conditions it is possible to add reagents in smaller increments than is possible with gravity fed devices.
9. A liquid/no-liquid decision by the computer requires five consecutive valve-pulse/detector-read sequences to be valid. This avoids problems with small bubbles which may form in the delivery line during prolonged additions with volatile solvents. It is essential that the tapered-bottom reservoir have its flat-cut-tipped eductor tube extending completely to the bottom. In this way all the liquid can be drawn through the tube before air bubbles become entrained in the flow.
10. The control programs are written in the C programming language and have been ported to IBM-PC (MSDOS) and CompuPro 8/16 (MSDOS and CP/M 80) computers. It is possible to write such programs so that they operate in a background mode, leaving the computer ostensibly free for other tasks.
11. Fox, R. W. and McDonald, A. T. (John Wiley and Sons, New York, 1985), p. 347.
12. Corey, E. J. and Nicolaou, K. C., *Journal of the American Chemical Society*, **96** (1974), 5614.
13 The reference lactone was prepared by meta-chloro perbenzoic acid oxidation of cyclododecanone ($CH_2Cl_2$, 25°, 72 h), standard work-up, and silica gel chromatography with 2% THF-hexanes.
14. Methyl purple is an acid-base indicator mixture containing an inert blue dye. The concentration of dye is adjusted so that the acid colour (pH <4·4) is purple, the base colour (pH >5·8) is green, and the neutral colour is a true grey.

### Appendix 1.A Electrical schematic of valve driver

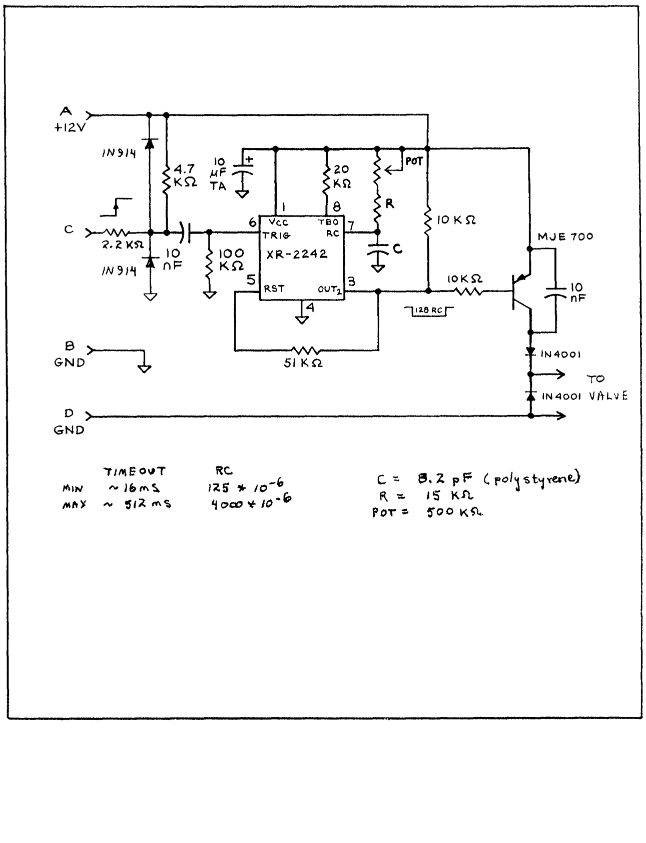

### Appendix 1.B Electrical schematic of liquid presence detector

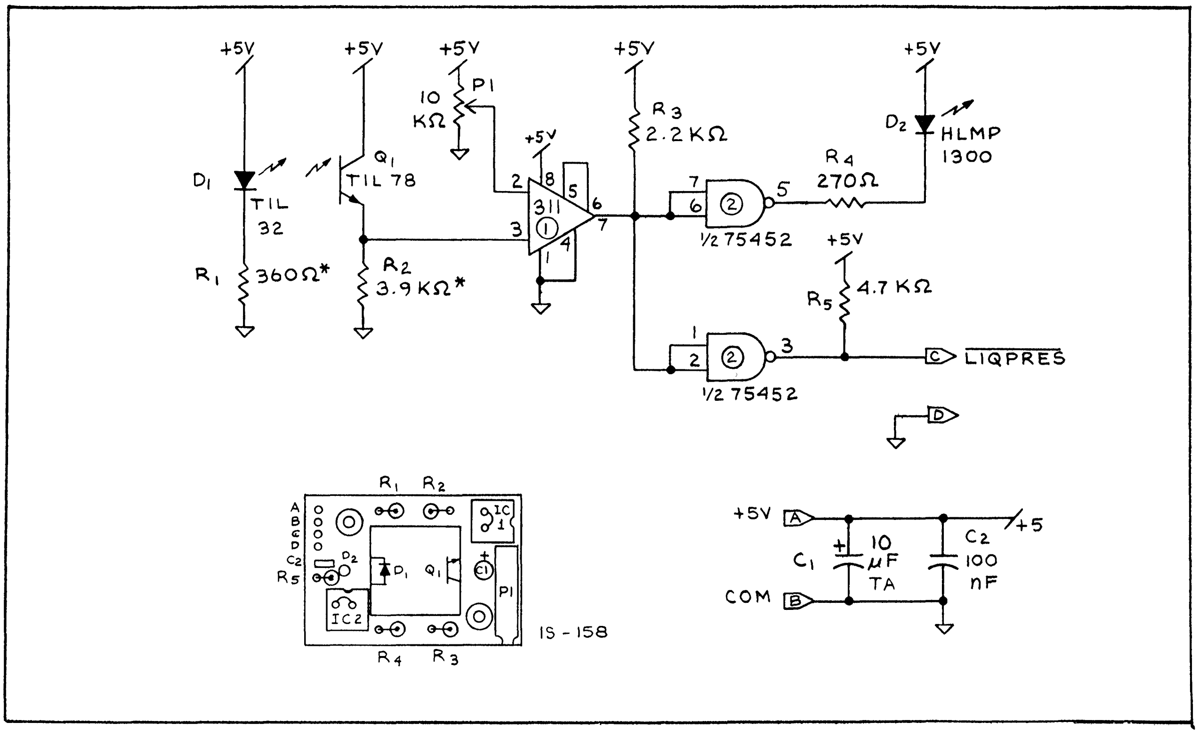

*CHOOSE FOR BEST RESPONSE

### Appendix 1.C Electrical schematic of optional RS 232 interface

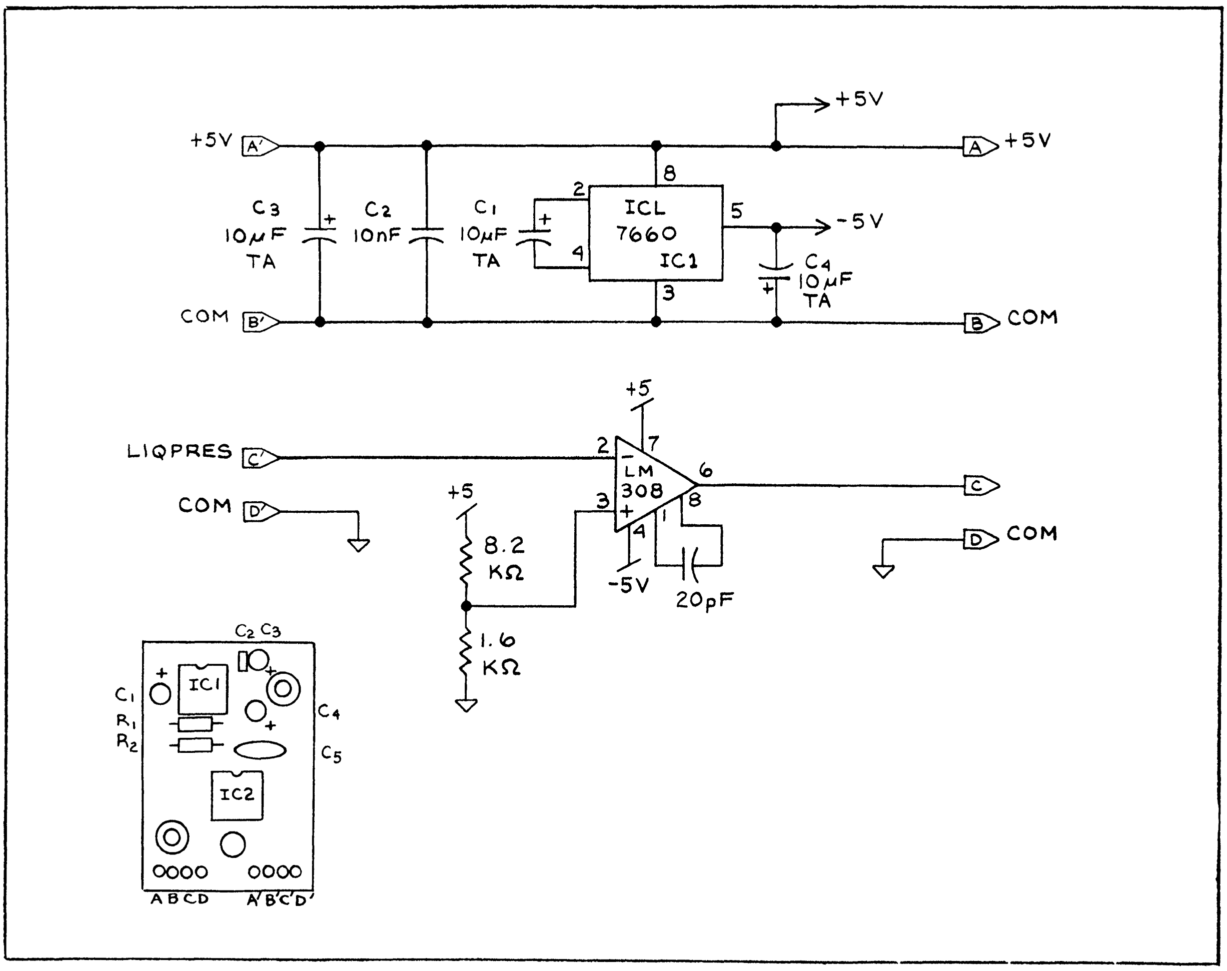

IS-159

### Appendix 1.D Mechanical specification of liquid presence detector

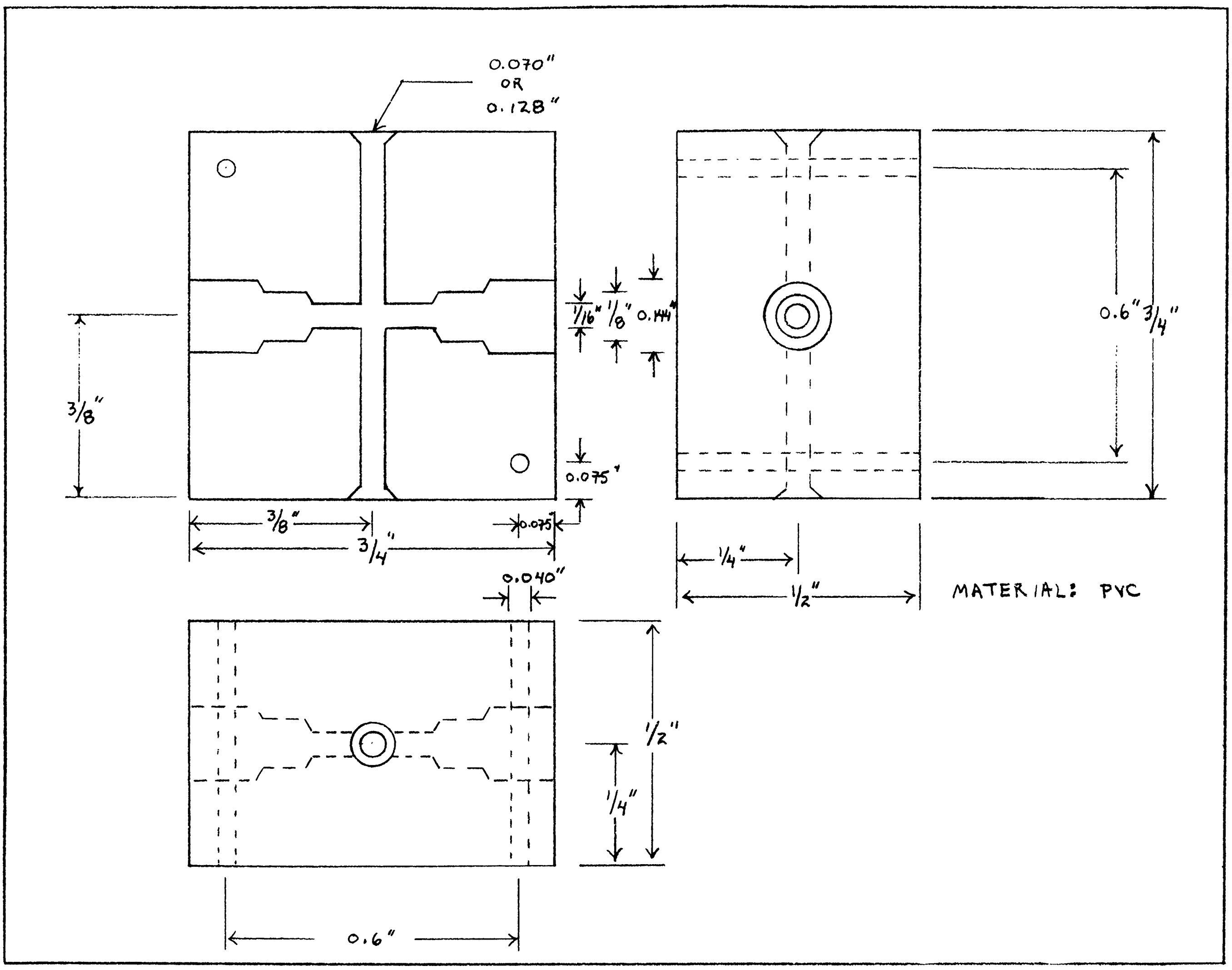

### Appendix 1.E Derivation of a simple liquid flow model

*Simple liquid flow model derivation*

*Basic assumptions*

(1) Laminar flow in horizontal tube

(2) Steady flow

(3) Incompressible fluid

(4) Fully developed flow

*Symbols and units*

$Q$: Flow in m³/s
$D$: Internal diameter of tube in m
$L$: Length of tubing in m
$\Delta P$: Pressure in Pa
$\mu$: Viscosity in poise (1 poise = 0.1 kg/ms)
$V_p$: Volume of liquid in tubing in m³
$V_d$: Volume of liquid delivered in m³.

$$Q = \frac{\pi \Delta P D^4}{128\,(0{\cdot}1\,\mu)\,L} \qquad (1)^1$$

and

$$Q = -\frac{dV_p}{dt} \qquad (2)$$

but $V_p = \pi\,(1/2\,D)^2\,L$

$$\text{or } L = \frac{4\,V_p}{\pi D^2} \qquad (3)$$

Substituting (3) in (1) gives:

$$-\frac{dV_p}{dt} = \frac{\pi^2 \Delta P D^6}{51{\cdot}2\,\mu\,V_p} \qquad (4)$$

$$\text{Let } K \text{ be } \frac{\pi^2 \Delta P D^6}{51.2\,\mu\,V_p} \qquad (5)$$

Substituting (5) in (4) and integrating gives:

$$-\int_{V_{p(0)}}^{V_{p(t)}} V_p\,dV = K \int_0^t t \qquad (6)$$

which gives: $V^2_{p(0)} - V^2_{p(t)} = (K/2)\,t$ (7)

with $V_{p(t)}$ = volume in tubing at time t

$V_{p(0)}$ = volume in tubing at time 0.

But $V_{p(0)} - V_{p(t)} = V_{d(t)}$

with $V_{d(t)}$ = volume delivered at time $t$.

$$\text{Or} \quad V_{p(t)} = V_{p(0)} - V_{d(t)} \tag{8}$$

Substituting (8) in (7) and developing gives:

$$V2d(t) - 2\,Vp(0)\,Vd(t) = -(K/2)\,t \tag{9}$$

*Application*

For the long system with water, we get:
$V_{p(0)} = 1\text{ ml} = 10^{-6}\text{ m}^3$ $\quad K = 1{\cdot}756\ 10^{-13}\text{ m}^6\text{ s}^{-1}$
Computed time required to drain the tube: 5·67 s.
Measured time required to drain the tube: (6 ± 0·1) s.

### Reference

1. Fox, R. W. and McDonald, A. T., *Introduction to Fluid Mechanics* (John Wiley and Sons, New York, 1985), 347.

### Appendix 1.F MS.DOS slow-adder control software listings

### Program pcrunadd.c

```
/* FILE: PCRUNADD.C */

/*
Desc: Main program to run the slow-adder from a PC
Date: 02/11/88
Vers: 1·00
Auth: JMH
*/

/*-----------------------------------

   C Compiler Used: Microsoft C Compiler Vers. 5·0
   Assembler Used: Microsoft Assembler Vers. 5·0

   Files required to link the program:

   pcrunadd.obj, pcadder.obj, pccal.obj, pcctrl.obj,
   pcaddio.obj, pcsleep.obj, dcd.obj, dtr.obj, clock.obj,
   delay.obj.

-----------------------------------*/

/*-----------------------------------

   The slow-adder is hooked up to the COM1 serial port
   of the PC.
   The program first calibrates the system, then performs
   the delivery according the user's run parameters and
   the calibration factor computed before.
   The program prints out the actual delivery time in
   minutes if it doesn't exceed the maximum delivery time
   (computed as required delivery time * 1·25). Other-
   wise, it prints out an error message.

   The volume to be delivered is a decimal number of ml,
   and the requested delivery time is given in minutes.
   (The smallest value advised is 10 min.) The program
   computes the minimum time allowed to deliver the
   requested volume and checks if the computed pulsing
   rate is actually smaller than 1 pulse/sec.

-----------------------------------

include "pcadder.h"

main()
{
int time, truetime, sladder();
long ftol(), mintime;
float calfact, vol, rate, slcalib();

/* Calibrate the slow-adder */

printf("\nEnter the calibration volume:");
scanf("%f", &vol); calfact = slcalib(vol);
printf ("\nCalibration factor = %f", calfact); if (calfact < 0)
      {
      printf("\nIncorrect calibration, job aborted");
      exit();
      }

/* Getting run parameters */

vol=time=0;
while (vol < = 0)
      {
      printf("\nEnter the volume to be delivered:");
      scanf("%F", &vol);
      if (vol <= 0)
            printf("\nIncorrect volume, try again");
      }
while (time <= 0)
      {
      /* Computes the minimum delivery time allowed*/
      mintime = ftol( (vol/MAXPULSE) / ( calfact*60.) );
      printf("\nMinimum time allowed = %ld min",
      mintime);
      printf("\nEnter the delivery time in min:");
      scanf("%d", &time);
      if(time<=0)
            printf("\nIncorrect time, try again");
      }

/* Checking run parameters validity */
/* Computes the actual pulsing rate & compares it
   with the */
/* maximum pulsing rate allowed ( 1 pulse / sec ) */

rate = ( (float) vol / ( 60 * (float) time) ) * calfact;
if (rate > MAXPULSE)
      {
      printf("\nPulsing rate exceeds slow-adder limits
      !!");
      exit();
      }

printf("START TIME"); /* Getting start time */
ptime();
```

```
if( (truetime = sladder( vol, time, calfact )) < 0)
        printf("\nDelivery incorrectly performed");
else
        printf("Actual addition time = %d
        minutes\n", truetime); printf("STOP TIME "); /* Getting stop time */
ptime();

}

/*-----------------------------------
Function ftol() : Converts a real into a long integer
-----------------------------------*/

ftol( number )
float number;
{
long value;
value = number;
return ( ( (number-val) < 0.5) ? value : value + 1)
}
```

### C routine pcadder.c

```
/* FILE : PCADDER */

/*
Desc: Main function to control the slow-adder
Date: 02/11/88
Vers: 2·00
Auth:    ARF/JMH
*/

/*-----------------------------------
    Function called as sladder( vol, time, calfact )

        float vol;      volume to be added
        float calfact;  calibration factor (ml/pulse)
        int time;       addition time in minutes

    returns PRIMERR : priming timeout
            ADDERR  : addition timeout (time > 1·25 *
            requested time)
            Addition time : addition correct
-----------------------------------*/

#include "pcadder.h"

sladder( vol, time, calfact)
int time;
float vol, calfact;
{
int err;
unsigned int delay;
long addtime, maxtime, sladd();

/* Computation of delay between 2 pulses expressed in
milliseconds */

delay = ftoi( ( ( 60 * (long) time ) / ( vol / calfact ) ) * 1000 );
printf ("\nDelay between 2 pulses = %ud ms", delay);
maxtime = 60 * (long) time * TIMEMULT : /* max
addtime 125% of calculated*/
printf("\nMaximum time allowed = %ld s", maxtime); if( err = slprime() )       /* PRIME SLOW ADDER */
    return( err );
if( (addtime = sladd( delay, maxtime )) < 0 )
                            /* ADD REAGENT */
return( (int)addtime ); slflush();                  /* MAKE SURE ALL HAS BEEN
                               FLUSHED OUT */
return( (int) (addtime/60) ); /* Returns addition time in
                                 min*/
}

/*-----------------------------------
    ftoi() – rounds floating point to the closest integer
                (not a standard C feature)
-----------------------------------

ftoi( num )
float num;
{
unsigned int val;
val=num;
return( ( (num-val) < ·5 ) ? val : val + 1 ) ;
}
```

### C routine pccal.c

```
/* FILE: PCCAL.C

Desc: Determines the calibration factor ( ml/pulse )
Date: 02/11/88
Vers: 2·00
Auth: ARF/JMH

*/

/*-----------------------------------

  Function called as slcalib(calvol)
float calvol : calibration volume (> 1ml for best results)

  After priming, pulses the slow-adder every second until
  no liquid is detected by the liquid detector. Computes
  the calibration factor as ml delivered / no of pulses.
  Requires 5 negative readings in a row to declare
  delivery complete.

Returns −1 if priming error (no liquid to sensor in 40
  pulses) or too many pulses required (maximum
  number of pulses = volume / fastest pulsing rate, i.e. 1
  pulse / sec), otherwise returns calibration factor in
  ml/pulse (drop size).

-----------------------------------

#include "pcadder.h"
```

```
float slcalib(calvol)
float calvol;
{

int pulses, maxpulse, i, err;
float cal,rate;
printf("\nBeginning calibration"); maxpulse = calvol / MINCALIB;          /* max pulses
                                          permitted */

if( err = slprime() )        /* PRIME TO SENSOR */
        return( (float)err );

/* Add until no liquid to sensor, pulsing rate = 1
   pulse/sec
   pulses = number of pulses
   pulseadder() = function to pulse the slow-adder once
   sleep(1000) = waits 1000 ms between 2 pulses */

   for( i=0, pulses=0 ; i < ADDOK ; pulseadder(),
               pulses++ sleep(1000) )
              if( pulses >= maxpulse )
                 return( ADDERR);
              else if( ! readsensor() )
                      i++;
              else
                      i=0; slflush();                    /* FLUSH OUT TUBING */

return((float) (calvol/(pulses+PRIMOK-ADDOK)));
}
```

**C routines pcctrl.c**

```
/* FILE:SLCTRL.C

Desc: Functions to prime, add a volume and flush the
      slow-adder
Date: 09/24/85
Vers: 1·00
Auth: ARF

*/

#include "pcadder.h"

/*------------------------------------

  * Function slprime() primes the slow-adder by
    pulsing liquid until 5 positive readings in a row from
    the liquid detector. Allows 40 pulses maximum.
    Returns 0 if ok, -1 if no liquid found.
  * Function sladd(delay, maxtime)

      unsigned int delay = delay between 2 pulses in ms
      long maxtime = maximum addition time
      (requested time * 1·25)

    Pulses the slow-adder until no liquid found and
    returns the elapsed time.
    Requires again 5 negative readings in a row to
    declare addition complete.

  * Function slflush() flushes the slow-adder by pulsing
    40 times with a pulsing rate of 1 pulse/sec.

--------------------------------------

/*------------------------------------
      slprime() - prime slow-adder for addition
------------------------------------*/

slprime()
{
int i, j; for( i=0,j=0 ; j < PRIMOK ; sleep( PRIMD ),
pulseadder(), i++)
     if(i > PRIMMAX-1)
        return( PRIMERR); /* error if no liquid found */
     else if( readsensor() )
        j++;  /* PRIMOK pos. reads before start of addn
     else
        j=0    /*if no liquid, reset index */

return(0);
}

/*------------------------------------
   sladd() - perform addition and return elapse time
------------------------------------*/

long sladd( delay, maxtime )
unsigned int delay;
long maxtime;
{
int i;
long starttime, gettime(), checktime(); starttime = gettime();        /* records starting time *

for( i=0 ; i <= ADDOK ; pulseadder(), sleep(delay) )
     if( maxtime <= checktime(starttime) )    /* check
                                           total time */
        return( ADDERR );
     else if( ! readsensor() )   /* increment on no liq. */
        i++;
     else                        /* if liquid reset index*/
        i=0; return( checktime( starttime ) );
}

/*------------------------------------
   slflush() - make sure all liquid is flushed from lines
------------------------------------*/

slflush()
}
int i; for( i=0; i < FLUSHNO; sleep( FLUSHD ), pulseadder(), i++ ) ;

}

```

### C routines pcaddio.c

```
/* FILE : PCADDIO.C

Desc: io routines for the slow-adder
Date: 09/10/85
Vers: 2·00
Auth: ARF/JMH

*/

#include "pcadder.h"

/*-----------------------------------------
        pulseadder() - pulse slow-adder once
-----------------------------------------*/
pulseadder()
}
dtr(1); /* toggle data terminal ready to pulse one-shot */
dtr(0);
}

/*-----------------------------------------
      readsensor() - reads the liquid detector
                    returns .1 if liquid, 0 if none
-----------------------------------------*/
readsensor()
{
if ( dcd() )        /* data carrier detect reads sensor */
     return( 0 );
return( 1 );
}

/*-----------------------------------------
ptime() - reads the PC real-time clock and prints time
-----------------------------------------*/
ptime()
{
int hr, min, sec;
time(&hr,&min,&sec);
printf("%02d:%02d:%02d\n",hr,min,sec);
}

/*-------------------------
gettime() - returns time in seconds
-------------------------*/
long gettime()
{
int hr, min, sec;
time(&hr,&min,&sec) ; return( (long)hr * 60 * 60 + min * 60 + sec );
}

/*-----------------------------------------
checktime() - returns time since start, allows clock to roll
-----------------------------------------*/
long checktime(starttime)
long starttime;
{
long time, gettime();
return( ( (time=gettime() ) >= starttime ) ?
     time-starttime:60L*60L*24L-starttime+time);
}
```

### C routine pcsleep.c

```
/* FILE: PCSLEEP.C

Desc: puts the system to sleep for a given time expressed
in milliseconds
Date: 02/12/88
Vers: 2·00
Auth: JMH

*/

sleep( time )
unsigned int time;
{
delay( time ); /* Delays the specified amount of
milliseconds */
}
```

**Assembly language routine pcdtr.asm**

```
; FILE : PCDTR.ASM
;
; Desc: toggle dtr according to param
; Date: 02/12/88
; Vers: 2·00
; Auth: ARF/JMH
;
;       synopsis        NULL dtr( tog )
;                       int tog;
;               tog=0, set logic low, else logic high

                ;
                .MODEL SMALL
                .CODE

                        PUBLIC_DTR

                _DTR    PROC
                        PUSH    BP              ; SAVE C'S BP
                ;
                ; === GET BASE ADDRESS OF COM1 FROM DATA AREA
                        MOV     AX,40H          ; POINT ES TO BIOS DATA AREA
                        MOV     ES,AX
                        MOV     DX,ES : [0]     ; GET COM1 BASE ADDRESS
                ;
                ; === GET PARAMETER
                        MOV     BP,SP           ; GET STACK PTR
                        MOV     AX,[BP+4]       ; READ PARAM
                ;
                ; === SELECT
                        CMP     AX,0            ; IS IT 0
                        JE      YES
                        MOV     AL,1            ; NO, SET TO ONE
                YES:
                ; === SWITCH DTR SIGNAL
                        ADD     DX,4            ; POINT TO MODEM CONTROL REG
                        OUT     DX,AL           ; SEND IT
                ;
                        POP     BP
                        RET

                _DTR    ENDP

                        END
```

**Assembly language routine pcdcd.asm**

```
; FILE : PCDCD.ASM
;
; Desc: Reads DCD signal from modem
; Date: 02/12/88
; Vers: 2·00
; Auth: ARF/JMH
                ;
                ;       SYNOPSIS
                ;               val = dcd( );
                ;               int val;
                ;       DESC – READS DCD RETURNING LOGIC 1 IF HIGH
                ;               LOGIC IF LOW
                ;
                ;
                .MODEL SMALL
                .CODE
```

```

        PUBLIC _DCD

_DCD    PROC

        PUSH    BP              ; SAVE C'S BP

; === GET COM1 ADDRESS

        MOV     AX,40H

        MOV     ES,AX

        MOV     DX,ES:[0]       ; OFFSET OF COM1 ADDRESS

        ADD     DX,6            ; POINT TO MODEM STATUS REG

        IN      AL,DX           ; GET THE VALUE

        AND     AL,80H          ; IS DCD SET?

        JZ      NO

        MOV     AX,1

        JMP     DONE

NO:     XOR     AX,AX

DONE:   POP BP

        RET

_DCD    ENDP

        END

```

### Assembly language routine pcclock.asm

```
; FILE: PCCLOCK.ASM
;
; Desc: Reads DOS clock and puts the vals in PTR
        locations
; Date: 02/12/88
; Vers: 2·00
; Auth: ARF/JMH
;
;    SYNOPSIS
;            NULL time( &hr, &min, &sec );
;            int hr,min,sec;
;            SMALL MEMORY MODEL ONLY
;

ARGS    EQU     4

   .MODEL SMALL
   .CODE

        PUBLIC _TIME

_TIME PROC

        PUSH    BP                      ; SAVE C'S BP

; === GET THE TIME

        MOV     AH,2CH                  ; DOS FUNCTION GETTIME

        INT     21H

; === GET ADDRESSES AND SAVE VALS

        MOV     BP,SP                   ; ADDRESSES FROM STACK

        MOV     BX, [BP+ARGS]           ; HOURS ADDRESS

        XOR     AH,AH                   ; CLEAR UPPER BYTE

        MOV     AL,CH                   ; SAVE HOURS

        MOV     [BX],AX

        MOV     BX, [BP+ARGS+2]         ; GET MIN. ADDRESS

        MOV     AL,CL

        MOV     [BX],AX

        MOV     BX, [BP+ARGS+4]         ; GET SECONDS ADDRESS

        MOV     AL,DH

        MOV     [BX],AX

DONE:   POP BP

        RET

_TIME ENDP

        END
```

### Assembly language routine pcdelay.asm

```
; Desc: Delay Function for Slow Adder Routines on a PC
; Date: 02/09/88
; Vers: 1·0
; Auth: GWK

; FILE: PCDELAY.ASM
;        02/09/88 GWK

;        SYNOPSIS – NULL _delay( count );
;                   int count;

;        DESC – sets up the sound timer as a 1 msec
;          counter
;          waits for count * 1 msec and returns –
;          delay is not precise due to TOD clock
;          interrupts

        PORTB   EQU     0061H        ;8255 PORT B I/O ADDRESS

        PORTC   EQU     PORTB + 1    ;8255 PORT C I/O ADDRESS

        CTC2    EQU     0042H        ;8253 CTC 2 I/O ADDRESS

        CTCMD   EQU     CTC2 + 1     ;8253 MODE I/O ADDRESS

          .MODEL SMALL
          .CODE
                PUBLIC _DELAY

        _DELAY PROC

                PUSH    BP           ;SAVE C'S BP REG

                MOV     BP, SP       ;GET ARGS FROM STACK

                MOV     DX, (BP+4]   ;GET COUNT INTO DX

                IN      AL, PORTB    ;SET UP 8255 PORT B

                OR      AL, 01H      ;SET BIT 0 HI TO ENABLE CTC2

                AND     AL, 0FDH     ;SET BIT 1 LO TO DISABLE SOUND

                OUT     PORTB, AL

                MOV     AL, 10110000B ;SET UP MODE FOR CTC2

                OUT     CTCMD, AL    ;CTC2, LSB/MSB, MODEO, BIN

        DELAY1: MOV     AX, 1193D    ;SET UP CTC2 DIVISOR FOR 1 MSEC

                OUT     CTC2, AL     ;SEND OUT LSB

                MOV     AL, AH       ;GET MSB

                OUT     CTC2, AL     ;SEND OUT MSB

        DELAY2: IN      AL, PORTC    ;READ IN 8255 PORT C

                AND     AL, 00100000B ;MASK FOR BIT 5

                JZ      DELAY2       ;JMP IF BIT 5 LOW

                DEC     DX           ;DECREMENT MSEC COUNTER

                JNZ     DELAY1       ;JMP IF NOT DONE

                POP     BP           ;RESTORE BP

                RET

          DELAY ENDP

                END
```

**Header file pcadder.h**

```
/* FILE: PCADDER.H

Desc: Header file for the slow-adder routines
Date: 02/12/88
Vers: 1·00
Auth: ARF/JMH

*/

#define PRIMMAX    40     /* max. pulses to prime
                             before returning error */
#define PRIMOK     5      /* min. positive readings
                             to confirm prime*/
#define PRIMERR    −1     /* error returned if can't
                             prime*/
#define PRIMD      1000   /* time between prime
                             pulses (msec.) */
#define ADDOK      5      /* min. positive readings
                             to confirm addn. end */
#define ADDERR     −2     /* error returned on
                             addition timeout */
#define FLUSHNO    5      /* number of pulses at end
                             to empty tubing */
#define FLUSHD     1000   /* time between flush
                             pulses (msec.) */
#define TIMEMULT   1·25   /* maximum addition
                             time before returning
                             error is calculated time
                             * TIMEMULT */
#define MINCALIB   0·001  /* Sets the minimum drop
                             size at 1 μl/pulse */
#define MAXPULSE   1      /* Sets the max pulse rate
                             to 1 pulse/s */
```

### Short courses

Loughborough University of Technology, UK, has announced the following short courses for 1989:

Fluorescence and Luminescence Spectrometry – 26–30 June 1989. Fee £480 including residence and all meals (£450 if paid with booking form). Non-residents £405 (£375).

Statistics for Analytical Chemistry – 11–14 July 1989. Fee £385 including residence and all meals (£355 if paid with booking form). Non-residents £325 (£295).

Flow Injection Analysis – 12–14 July 1989. Fee £325 including residence and all meals (£300 if paid with booking form). Non-residents £275 (£250).

*For further details please contact: Mrs J. E. Stirling, Department of Chemistry, Loughborough University of Technology, Loughborough, Leics. LE11 3TU. Telephone: (0509) 222549.*

HPLC Technology and Applied Chromatography Systems are holding five HPLC Beginners Training Courses during 1989. Each course will last three days and will include both practical and discussion sessions. The courses are held at The Deanwater Hotel, Woodford, Cheshire. All purchasers of HPLC Systems from ACS receive a complimentary place on the course.

*For further information please contact: Applied Chromatography Systems, The Arsenal, Heapy Street, Macclesfield, Cheshire SK11 7JB. Telephone: (0625) 34575.*

Chemserve have announced two courses for 1989:

Fluorine Spectroscopy Workshop – 10–11 April 1989.

Mass Spectrometry for Beginners – 17–18 April 1989.

*For further information, please contact: Chemserve, UMIST, PO Box 88, Manchester M60 1QD. Telephone: 061 228 7700.*

The University of Liverpool is holding a short course on Modern Spectroscopic Techniques, 2–7 April 1989.

*For further information, please contact: Dr A. Hodgson, Dept. of Chemistry, University of Liverpool, PO Box 147, Liverpool L69 3BX.*

Royal Society of Chemistry Residential School – 28–31 March 1989. Computer Methods in UV, vis and ir Spectroscopy, Polytechnic of Wales.

*For further information, contact Ms L. Hart, RSC, 30 Russell Square, London WC1B 5DT. Telephone: 01-631-1355.*

